# Job burnout and work-family conflict among emergency nurses in China: a cross-sectional study based on the job demands-resources model

**DOI:** 10.3389/fpubh.2026.1869989

**Published:** 2026-06-26

**Authors:** Xiaoxiao Chen, Hao Zhang, Min Dai, Xiaoli Chen, Yongli Gao

**Affiliations:** 1 Department of Emergency Medicine, West China Hospital, Sichuan University/West China School of Nursing, Sichuan University, Chengdu, China; 2 Institute of Disaster Medicine, Sichuan University, Chengdu, China; 3 Nursing Key Laboratory of Sichuan, Chengdu, China

**Keywords:** correlation analysis, emergency department nurses, job burnout, occupational health, work-family conflict

## Abstract

**Background:**

Job burnout and work-family conflict have become prevalent occupational health issues in the healthcare industry. Emergency department (ED) nurses face dual pressures from high-intensity work environments and multiple role responsibilities, making them highly susceptible to job burnout. This study aimed to describe the current status of job burnout and work-family conflict among ED nurses, and explore their correlation as well as the associated factors of job burnout, so as to provide empirical evidence for formulating targeted occupational health intervention measures for ED nurses.

**Objective:**

Based on the Job Demands-Resources (JD-R) model, this study investigated the current status of job burnout and work-family conflict among ED nurses, and analyzed the correlational relationship between work-family conflict and job burnout, intending to provide evidence-based references for improving the occupational health status of emergency nursing staff.

**Methods:**

A cross-sectional survey was conducted from December 26, 2023–January 18, 2024. A total of 1,540 ED nurses from 30 hospitals were enrolled in the study. Data were collected via a self-designed sociodemographic questionnaire and two standardized scales for assessing job burnout and work-family conflict. Descriptive statistics, Pearson correlation analysis, and multiple linear regression analysis were performed using SPSS 26.0 (IBM Corporation, Armonk, New York, United States) to analyze the correlational relationships among variables.

**Results:**

All 1,540 distributed questionnaires were valid for analysis. Most participants were female, held a bachelor's degree, and had children. The average total score of job burnout was 4.77 (SD = 6.16), with subscale scores of 11.30 (SD = 7.76) for emotional exhaustion, 8.61 (SD = 7.58) for cynicism, and 13.79 (SD = 10.04) for professional efficacy. The average total score of work-family conflict was 42.48 (SD = 16.21), including 18.27 (SD = 6.88) for work-to-family conflict and 24.21 (SD = 10.75) for family-to-work conflict. Correlation analysis showed that the total score and the scores of the two dimensions of work-family conflict were positively correlated with the total score and the scores of all three dimensions of job burnout (*r* = 0.493, 0.553, 0.389; all *P* < 0.001). Multiple linear regression analysis identified significant associated factors of job burnout, including parental status (*B* = −1.126, 95% CI: −2.041 to −0.211, *P* = 0.016), weekly working hours of 49–58 h (*B* = 1.010, 95% CI: 0.005–2.015, *P* = 0.049), weekly working hours of ≥ 59 h (*B* = 2.503, 95% CI: 1.128–3.878, *P* < 0.001), monthly night shift frequency of ≥ 9 times (*B* = 1.324, 95% CI: 0.344–2.303, *P* = 0.008), and work-to-family conflict (*B* = 0.449, 95% CI: 0.397–0.501, *P* < 0.001).

**Conclusions:**

Long working hours and frequent night shifts are closely correlated with aggravated emotional exhaustion, and work-family role conflict is significantly associated with higher job burnout levels among ED nurses. Hospital administrators should formulate targeted intervention strategies, including reasonably regulating working hours, reducing excessive night shifts, and building a family-supportive organizational atmosphere, to alleviate job burnout and stabilize the emergency nursing workforce.

## 1 Introduction

Job burnout is a psychological syndrome characterized by emotional exhaustion, cynicism, and reduced professional efficacy, which occurs when frontline service staff fail to cope with long-term work stress effectively (1). Medical and nursing staff are exposed to persistent occupational stress, resulting in a high prevalence of job burnout worldwide. A recent umbrella review demonstrated that approximately 30.7% of global nurses suffer from job burnout, with the pooled prevalence of emotional exhaustion, depersonalization, and low personal accomplishment reaching 33.45%, 25.0%, and 33.49%, respectively (2). The emergency department is a high-stress workplace with unpredictable work tasks, intensive emergency treatments, continuous heavy workloads, and tense nurse-patient relationships (3, 4). ED nurses not only undertake high-intensity daily work and cope with fluctuating patient volumes but also frequently encounter traumatic resuscitation events, workplace violence risks, and circadian rhythm disorders caused by rotating shifts, while facing the psychological pressure of life-and-death medical scenarios (5–7). These cumulative occupational stresses make ED nurses a high-risk group for job burnout.

In the United States, more than half of medical staff experience job burnout symptoms during their careers (8). Although the prevalence of burnout varies across medical specialties, emergency medicine practitioners consistently show the highest burnout levels (9). The burnout rate of medical staff ranges from 30.9 to 88.0% across different job positions (10–13), and ED staff exhibit significantly higher burnout levels than other medical occupational groups. A large-scale cross-sectional survey of 20,136 Chinese ED nurses conducted in 2023 reported a burnout prevalence of 87.6%, with 34.1% of participants experiencing severe burnout; all dimensional scores of burnout were higher than those reported in international studies conducted during the same period (14). Accordingly, job burnout among ED nurses deserves extensive attention and targeted intervention.

Work-family conflict refers to the role conflict formed when work demands hinder the fulfillment of family responsibilities or family burdens interfere with normal work performance (15). The special occupational attributes of ED nursing restrict nurses' ability to perform family roles, bringing long-term difficulties in undertaking housework, participating in child rearing, and maintaining intimate family relationships. Previous studies have confirmed the high prevalence of work-family conflict among nurses. Pien et al. (16) found that work-family conflict is common and persistent among nursing staff; Zandian et al. (17) further reported that up to 93% of nurses experience moderate to severe work-family conflict. A large-scale survey of 70,932 Chinese nurses conducted by Chen et al. (18) also verified that work-family conflict has become a prominent occupational dilemma for domestic nurses.

The Job Demands-Resources (JD-R) model provides a mature theoretical framework for explaining occupational stress and burnout. In this framework, work-family conflict is defined as a negative job demand that consumes individual psychological resources and induces emotional exhaustion (19). Existing observational studies have confirmed a significant positive correlation between work-family conflict and nurse job burnout, and this correlation remains stable after controlling for workload variables (20). In addition, recent high-quality studies have shown that effort-reward imbalance is prevalent among ED nurses (26.2%), and long working hours (≥59 h/week) and overcommitment are key associated factors of occupational stress (21). The above findings indicate that structural workload pressure and work-family conflict are jointly associated with elevated burnout risk among ED nurses.

Nevertheless, existing studies on ED nurses' burnout mainly focus on internal workplace stressors, while the correlational relationship between work-family conflict and burnout has not been fully explored and systematically analyzed. To fill this research gap, this study incorporated work-family conflict into the analytical framework to explore the associated factors of ED nurses' job burnout, so as to provide a theoretical basis for developing intervention strategies combining occupational support and family-friendly management. It is worth emphasizing that this study adopts a cross-sectional design and cannot verify causal relationships or mediating mechanisms. The JD-R model is only used as a theoretical perspective to interpret the observed correlational relationships between variables.

## 2 Methods

### 2.1 Study design and participants

This study adopted a multicenter cross-sectional research design and was carried out from December 26, 2023, to January 18, 2024. Participants were recruited via stratified cluster sampling. Based on the seven major geographical divisions of China (Northeast, North, East, Central, South, Southwest, and Northwest China), 2–3 representative tertiary hospitals were selected from provincial capitals, prefecture-level cities, and municipalities directly under the Central Government in each region. All eligible ED nurses in the selected hospitals were included in the study.

Inclusion criteria: (1) holding a valid and registered nursing practicing certificate; (2) having worked continuously in the emergency department for more than one year; (3) voluntary participation in the study with signed informed consent. Exclusion criteria: (1) nurses receiving standardized residency training; (2) nurses on further study and training; (3) nurses on sick leave, maternity leave, or other long-term absence during the investigation period.

### 2.2 Measurement tools

#### 2.2.1 Demographic questionnaire

A self-designed sociodemographic questionnaire was used to collect participants' basic information, including age, gender, educational background, marital status, and parental status.

#### 2.2.2 Job burnout scale

Job burnout was measured using the Chinese revised version of the Maslach Burnout Inventory–General Survey (MBI-GS). The original scale was developed by Maslach et al. (1), and the Chinese revised version translated and revised by Li and Shi (22) has been widely applied in domestic occupational population research with good reliability and validity. The scale consists of 16 items covering three dimensions: emotional exhaustion (5 items), depersonalization (4 items), and professional efficacy (6 items).

Emotional exhaustion and depersonalization are positively scored, with higher scores indicating more severe burnout. The original professional efficacy dimension is positively scored (higher scores represent better professional efficacy). To unify the scoring direction (higher scores indicating higher burnout levels), each item of the professional efficacy dimension was reverse-scored (i.e., 6 minus the original score), and the reversed scores were summed to obtain a reversed total score. A comprehensive burnout composite score was calculated in accordance with the weighting formula proposed by Li and Shi (22): composite burnout score = 0.4 × total emotional exhaustion score + 0.3 × total depersonalization score + 0.3 × reversed professional efficacy total score.

#### 2.2.3 Work–family conflict scale

The Work–Family Behavioral Role Conflict Scale (WFBRC-S) developed by Clark et al. (23) and revised by Zhang (24) was used to assess nurses' work-family conflict. The scale contains 19 items divided into two dimensions: work-to-family behavioral role conflict and family-to-work behavioral role conflict. All items adopt a 5-point Likert scoring method, with higher total and dimensional scores indicating more serious work-family conflict. In this study, the Cronbach's α coefficient of the scale was 0.969, indicating excellent internal consistency reliability.

### 2.3 Data collection

In this study, all data were collected through an online questionnaire designed and published on the Wenjuanxing platform (https://www.wjx.cn/). The first page of the electronic questionnaire displayed a detailed informed consent statement, including the research purpose, voluntary participation principle, anonymous survey setting, data confidentiality commitment, and the right of participants to withdraw from the survey at any time without adverse consequences. Participants could only access the formal questionnaire items after checking the option of “I have read and agree to the informed consent.”

With the coordination of the Emergency Nursing Committee of the Chinese Nursing Association, the questionnaire QR code and research introduction were distributed to ED nurse WeChat work groups of all participating hospitals. Eligible nurses participated in the survey voluntarily. To ensure data quality, the platform was set to allow only one submission per IP address to avoid repeated responses. After data collection, logical screening was conducted to eliminate invalid questionnaires with full consistent answers, contradictory basic information, and illogical filling content.

### 2.4 Statistical analysis

SPSS 26.0 statistical software was used for data analysis. Continuous variables conforming to normal distribution were described as mean ± standard deviation (SD), and categorical variables were expressed as frequencies and percentages. Independent-samples t-test and one-way Analysis of Variance (ANOVA) were used for inter-group comparison of continuous variables. Pearson correlation analysis was adopted to analyze the correlational relationships between job burnout (total and dimensional scores) and work-family conflict (total and dimensional scores).

On the basis of univariate analysis, multiple linear regression analysis was performed. The total burnout score was taken as the dependent variable, work-family conflict indicators as independent variables, and statistically significant sociodemographic and work characteristic indicators in univariate analysis as covariates. Variance inflation factor (VIF) was used to test multicollinearity, with VIF < 5 indicating no serious multicollinearity. A two-tailed *P* < 0.05 was considered statistically significant.

### 2.5 Ethical considerations

This study involved human participants and was approved by the Ethics Review Committee of West China Hospital of Sichuan University (Approval No.: 2024.309). All participants were fully informed of the research content and signed informed consent before participation, and the survey process followed the principles of voluntariness, anonymity, and confidentiality.

## 3 Results

### 3.1 Basic characteristics of participants

A total of 1,540 valid questionnaires were collected, covering ED nurses from 30 tertiary general hospitals in seven geographical regions of China. The average age of participants was 32.23 ± 6.80 years old. Among all participants, 78.6% were female, 63.6% were married, and 32.0% had 6–10 years of working experience. In terms of work characteristics, 50.6% of nurses worked 41–48 hours per week, and 42.8% had 5–8 night shifts per month. Detailed sociodemographic and work-related characteristics are shown in Table 1.

**Table 1:** Characteristics of the participants (*n* = 1,540).

Variables	Categories	*n*	%
Age	20–29 years	582	37.8
	30–39 years	734	47.7
	40–49 years	183	11.9
	≥50 years	41	2.7
Gender	Female	1,211	78.6
	Male	329	21.4
Marital status	Unmarried	560	36.4
	Married	980	63.6
Number of children	No	710	46.1
	Yes	830	53.9
Educational level	Associate degree	186	12.1
	Bachelor's degree or above	1,354	87.9
Professional title	Junior RN	907	58.9
	Middle RN	574	37.3
	Senior RN	59	3.8
Years of nursing experience	≤ 5years	491	31.9
	6–10 years	493	32.0
	11–15 years	300	19.5
	16–20 years	121	7.8
	>20 years	135	8.8
Working hours per week	≤ 40 h per week	577	37.5
	41~48 h per week	780	50.6
	49~58 h per week	123	8.0
	≥59 h per week	60	3.9
Night shift	No	181	11.8
	Yes	1,359	88.2
Number of night shift	0	181	11.8
	1–4 times per month	239	15.5
	5–8 times per month	659	42.8
	>8 times per month	461	29.9
Monthly income (yuan)	< 4,000 yuan	57	3.7
	4,000–5,999 yuan	149	9.7
	6,000–7,999 yuan	250	16.2
	8,000–9,999 yuan	368	23.9
	≥10,000 yuan	716	46.5
Smoking status	No	1,453	94.4
	Yes	87	5.6
Drinking status	No	1,355	88.0
	Yes	185	12.0

RN, Registered nurse.

### 3.2 Descriptive analysis of job burnout and work-family conflict scores

The total and dimensional scores of job burnout and work-family conflict all conformed to normal distribution. The dimensional scores of job burnout were 11.30 ± 7.76 for emotional exhaustion, 8.61 ± 7.58 for cynicism, and 13.79 ± 10.04 for professional efficacy. The dimensional scores of work-family conflict were 18.27 ± 6.88 for work-to-family conflict and 24.21 ± 10.75 for family-to-work conflict. Detailed descriptive statistical results are presented in Table 2.

**Table 2:** Analysis of job burnout and work-family conflict scores.

Variables	Dimension	Min	Max	Mean	SD
Job burnout	Emotional exhaustion	0	30	11.30	7.76
	Cynicism	0	30	8.61	7.58
	Personal accomplishment	0	36	13.79	10.04
	Overall score	−9	22.8	4.77	6.16
Work-to-family role conflict	Work-to-family conflict	7	35	18.27	6.88
	Family-to-work conflict	12	60	24.21	10.75
	Total scores	19	95	42.48	16.21

SD, standard deviation.

### 3.3 Correlation analysis between job burnout and work-family conflict

Pearson correlation analysis showed that the total score and each dimensional score of work-family conflict were significantly positively correlated with the total score and three-dimensional scores of job burnout (all *P* < 0.01). Specifically, emotional exhaustion was strongly positively correlated with cynicism (*r* = 0.878, *P* < 0.01), and moderately and strongly positively correlated with work-to-family conflict (*r* = 0.642, *P* < 0.01) and family-to-work conflict (*r* = 0.510, *P* < 0.01). The total score of work-family conflict was moderately positively correlated with the total burnout score (*r* = 0.493, *P* < 0.01). Detailed correlation results are shown in Table 3.

**Table 3:** Pairwise correlation analysis of total scores and sub-dimensions between job burnout and work-family conflict.

Variables	Job burnout				Work-to-family role conflict		
	Emotional exhaustion	Cynicism	Personal accomplishment	Overall score	Work-to-family conflict	Family-to-work conflict	Total scores
Emotional exhaustion	1						
Cynicism	0.878^**^	1					
Personal accomplishment	−0.096^**^	0.013	1				
Overall score	0.876^**^	0.806^**^	−0.533^**^	1			
Work-to-family conflict	0.642^**^	0.606^**^	−0.012	0.553^**^	1		
Family-to-work conflict	0.510^**^	0.561^**^	0.154^**^	0.389^**^	0.676^**^	1	
Total scores	0.610^**^	0.629^**^	0.097^**^	0.493^**^	0.873^**^	0.950^**^	1

^**^P < 0.01.

## 4 Associated factors of job burnout

### 4.1 Univariate analysis

Univariate analysis was conducted with job burnout total score as the dependent variable and sociodemographic indicators and work-family conflict levels as independent variables. The results showed that age, marital status, parental status, educational background, professional title, working years, weekly working hours, and monthly night shift frequency were significantly associated with job burnout (all *P* < 0.05), as detailed in Table 4.

**Table 4:** Results of univariate analysis of job burnout.

Variables	Categories	x±s	*t*/F	*P*
Age	20–29 years	3.84 ± 6.47	8.462	< 0.001
	30–39 years	5.16 ± 6.00		
	40–49 years	6.12 ± 5.46		
	≥50 years	4.77 ± 5.39		
Gender	Female	4.49 ± 6.80	0.857	0.392
	Male	4.84 ± 5.97		
Marital status	Unmarried	4.17 ± 6.47	−2.892	0.004
	Married	5.11 ± 5.95		
Number of children	No	4.41 ± 6.33	−2.090	0.037
	Yes	5.07 ± 5.99		
Educational level	Associate degree	2.89 ± 6.53	−4.467	< 0.001
	Bachelor's degree or above	5.02 ± 6.06		
Professional title	Junior RN	4.08 ± 6.30	15.614	< 0.001
	Middle RN	5.60 ± 5.78		
	Senior RN	7.16 ± 6.03		
Years of nursing experience	≤ 5years	3.74 ± 6.30	6.126	< 0.001
	6–10 years	4.94 ± 6.27		
	11–15 years	5.19 ± 5.93		
	16–20 years	5.90 ± 5.73		
	>20 years	5.89 ± 5.60		
Working hours per week	≤ 40 h per week	3.77 ± 5.80	13.455	< 0.001
	41~48 h per week	5.02 ± 5.85		
	49~58 h per week	6.31 ± 7.72		
	≥59h per week	7.85 ± 7.75		
Night shift	No	4.78 ± 6.18	0.223	0.823
	Yes	4.67 ± 6.00		
Number of night shift	0	4.67 ± 6.00	7.242	< 0.001
	1–4 times per month	3.46 ± 5.53		
	5–8 times per month	4.62 ± 6.17		
	>8 times per month	5.69 ± 6.38		
Monthly income (yuan)	< 4,000 yuan	6.06 ± 7.90	1.057	0.377
	4,000–5,999 yuan	4.54 ± 6.93		
	6,000–7,999 yuan	4.76 ± 6.80		
	8,000–9,999 yuan	4.41 ± 6.18		
	≥10,000 yuan	4.89 ± 5.55		
Smoking status	No	4.73 ± 6.05	−0.645	0.521
	Yes	5.28 ± 7.76		
Drinking status	No	4.70 ± 6.12	−1.117	0.264
	Yes	5.24 ± 6.45		

### 4.2 Multivariable analysis

#### 4.2.1 Collinearity diagnostics

All variables with statistical significance in univariate analysis and two dimensional indicators of work-family conflict were included in the multiple linear regression model. Collinearity diagnosis results showed that the tolerance of all variables was greater than 0.1 and VIF values were all lower than 10, indicating no serious multicollinearity among variables (Supplementary Table S1).

#### 4.2.2 Multiple linear regression results

Multiple linear regression analysis demonstrated that parental status, weekly working hours (49–58 h/week, ≥59 h/week), monthly night shift frequency (≥9 times/month), and work-to-family conflict were significant associated factors of ED nurses' job burnout (all *P* < 0.05). The regression equation was as follows: Composite burnout score = −5.995 – 1.126 × parental status (having children) + 1.010 × weekly working hours (49–58 h) + 2.503 × weekly working hours (≥59 h) + 1.324 × monthly night shift frequency (≥9 times) + 0.429 × work-to-family conflict score.

Specifically, nurses with children had a 1.126-point lower burnout composite score than those without children. Compared with nurses working ≤ 40 hours per week, nurses working 49–58 h and ≥59 h per week had 1.010-point and 2.503-point higher burnout scores, respectively. Nurses with ≥9 night shifts per month had a 1.324-point higher burnout score than those with fewer night shifts. Each one-point increase in work-to-family conflict score was associated with a 0.429-point increase in burnout composite score (Table 5).

**Table 5:** Results of multiple linear regression analysis.

Variables		Unstandardized coefficient		Standardized coefficient	*t*	*P*	95%CI	
		*B*	*SE*	*Beta*			Lower	Upper
Constant		−5.995	0.673		−8.902	< 0.001	−7.316	−4.674
Age	20–29 years	0						
	30–39 years	0.571	0.555	0.046	1.028	0.304	−0.518	1.660
	40–49 years	0.645	0.940	0.034	0.686	0.493	−1.198	2.488
	≥50 years	0.061	1.342	0.002	0.046	0.964	−2.570	2.693
Marital status	Unmarried	0						
	Married	0.319	0.467	0.025	0.683	0.495	−0.597	1.235
Number of children	No	0						
	Yes	−1.126	0.466	−0.091	−2.414	0.016	−2.041	−0.211
Educational level	Associate degree	0						
	Bachelor's degree or above	0.749	0.417	0.040	1.798	0.072	−0.068	1.566
Professional title	Junior RN	0						
	Middle RN	0.485	0.350	0.038	1.388	0.165	−0.200	1.171
	Senior RN	1.221	0.808	0.038	1.512	0.131	−0.363	2.806
Years of nursing experience	≤ 5 years	0						
	6–10 years	−0.143	0.541	−0.011	−0.264	0.792	−1.205	0.919
	11–15 years	−0.400	0.674	−0.026	−0.593	0.553	−1.723	0.923
	16–20 years	0.233	0.888	0.010	0.262	0.793	−1.508	1.974
	>20 years	1.303	1.116	0.060	1.168	0.243	−0.886	3.492
Working hours per week	≤ 40 h per week	0						
	41~48 h per week	0.445	0.282	0.036	1.577	0.115	−0.109	0.999
	49~58 h per week	1.010	0.513	0.044	1.971	0.049	0.005	2.015
	≥59 h per week	2.503	0.701	0.079	3.570	< 0.001	1.128	3.878
Number of night shift	0	0						
	1–4 times per month	0.224	0.540	0.013	0.415	0.678	−0.836	1.284
	5–8 times per month	0.888	0.481	0.071	1.845	0.065	−0.056	1.832
	>8 times per month	1.324	0.499	0.098	2.650	0.008	0.344	2.303
Work-to-family role conflict	Work-to-family conflict	0.449	0.026	0.502	16.989	< 0.001	0.397	0.501
	Family-to-work conflict	0.020	0.017	0.036	1.223	0.221	−0.012	0.053

#### 4.2.3 Significance test of the regression model

ANOVA test of the regression model showed *F* = 37.104, *P* < 0.001, indicating that the overall regression model was statistically significant, and the included independent variables could effectively explain the variation of job burnout (Supplementary Table S2).

#### 4.2.4 Model fit test

The model fit results showed that the adjusted *R*^2^ was 0.319, indicating that the model could explain 31.9% of the total variation of ED nurses' job burnout. The Durbin–Watson statistic was 1.928, close to 2, suggesting no autocorrelation in residual errors and good model fitting validity (Supplementary Table S3).

## 5 Discussion

Based on the JD-R theoretical framework, this multicenter cross-sectional study systematically analyzed the correlations among working intensity (weekly working hours, night shift frequency), work-family conflict, parental status, and job burnout among Chinese ED nurses. The research results confirm that work-family conflict is significantly positively correlated with emotional exhaustion and cynicism, which is consistent with the findings of most occupational health studies (25–27). On the basis of verifying existing research conclusions, this study supplements empirical evidence of the correlation between work stress and burnout among Chinese ED nurses, and further analyzes the differential correlation of different dimensions of work-family conflict with burnout.

This study has three main strengths. First, it adopts a large-sample and multicenter research design, providing targeted data support for occupational health research of Chinese ED nurses, who are underrepresented in international studies. Second, this study verifies the applicability of the JD-R model in explaining the occupational stress-burnout correlation in high-pressure emergency nursing scenarios. Third, the finding of lower burnout levels among nurses with children provides a new perspective for exploring the dual attributes of family roles (work demand and psychological resource) (28, 29). It should be emphasized that this study only observed a correlational relationship, and relevant conclusions need to be further verified by longitudinal studies.

This study found that work-to-family conflict was the most prominent associated factor of job burnout (*B* = 0.449, 95% CI: 0.397–0.501, β = 0.502, *P* < 0.001), while family-to-work conflict had no significant correlation with burnout (*B* = 0.020, *P* = 0.221). This indicates that for ED nurses, the work-to-family role interference is more closely associated with occupational burnout than family-to-work interference. Long working hours and frequent night shifts also showed significant positive correlations with burnout. Compared with nurses working standard hours, nurses with excessive working hours and frequent night shifts face more severe time and energy consumption, which makes it difficult to balance family responsibilities and further aggravates work-family role conflict (30, 31). Continuous role conflict consumes nurses' psychological and emotional resources, ultimately leading to aggravated emotional exhaustion and cynicism, and increased burnout levels (27, 32).

Notably, working hours, night shift frequency, and work-family conflict have conceptual overlapping attributes. Excessive working intensity may be correlated with burnout both directly and indirectly through increased work-family conflict. Although the VIF values of all variables are within the reasonable range, the overlapping connotation of predictive variables suggests that the regression coefficients may reflect shared variable variation rather than completely independent effects. Future longitudinal studies can further explore the potential mediating and moderating effects among variables to clarify the complex correlational relationships between workload, work-family conflict, and burnout.

After controlling for confounding variables, this study found that nurses with children had significantly lower burnout scores. This counter-intuitive result cannot be simply interpreted as the protective effect of family resources. Restricted by the cross-sectional design, this result may be affected by multiple confounding factors such as selection bias, age maturity effect, and survivor bias. It is also impossible to rule out reverse correlation: nurses with lower burnout levels may have more stable family relationships and are more capable of undertaking parental roles. In addition, this study did not collect data on family support level, household labor division, and parenting quality, so the potential influencing factors behind this correlation cannot be further analyzed (28, 33). Therefore, this result is only exploratory and needs further verification by subsequent longitudinal and in-depth research.

Job burnout among ED nurses will induce individual negative psychological problems such as anxiety and sleep disorders, and also increase the risk of nurse turnover and adverse patient events, which is not conducive to the stable development of emergency nursing teams (34–38). Based on the correlational characteristics of relevant variables, hospital management should optimize intervention strategies for nurse burnout. In addition to appropriately controlling working hours and reducing unreasonable night shifts, it is necessary to focus on alleviating work interference with family life, build family-supportive organizational culture, and provide flexible scheduling and humanistic care services (39, 40). For parental nurses, targeted support measures should be formulated to avoid work-family conflict offsetting the potential protective effect of family resources.

The research samples of this study are all from tertiary general hospitals with high work intensity and standardized emergency service procedures. Therefore, the research conclusions are not fully applicable to primary hospitals, community medical institutions, and rural medical settings. Future research can expand the sample scope and adopt longitudinal tracking design to further explore the correlational characteristics of burnout-related variables.

### 5.1 Limitations

This study has several limitations. First, the cross-sectional design can only analyze the correlational relationships between variables and cannot determine causal direction or verify potential mediating and moderating mechanisms. Second, the samples are limited to tertiary general hospitals, which reduces the population representativeness of the conclusions. Third, all data are self-reported questionnaire data collected at a single time point, which may cause common method variance (CMV) and overestimate the correlation coefficients between variables, although procedural control measures such as anonymous filling and scattered item setting have been adopted. Fourth, this study did not measure family support, household structure, and parenting status, failing to further explain the correlation between parental status and burnout. Finally, the interactive effect of parental status and work-family conflict on burnout needs further verification.

## 6 Conclusion

Significant positive correlations exist between work-family conflict and job burnout among Chinese ED nurses, among which work-to-family conflict is most closely correlated with emotional exhaustion and cynicism. Long working hours and frequent night shifts are important work stressors closely associated with increased burnout levels, which may further interact with work-family conflict to aggravate resource consumption and occupational burnout. Having children is correlated with lower burnout levels among ED nurses, but this exploratory result needs further longitudinal verification. Hospital administrators should optimize working schedule management, reduce excessive working hours and night shift burden, and implement family-friendly management policies to alleviate work-family conflict and relieve nurse burnout. Future studies should adopt longitudinal designs and multi-source objective measurement indicators to further clarify the complex correlational relationships among relevant variables and provide more accurate evidence for formulating burnout intervention strategies.

## Data Availability

The original contributions presented in the study are included in the article/Supplementary material, further inquiries can be directed to the corresponding author.
